# Comprehensive Vitamer Profiling of Folate Mono- and Polyglutamates in Baker’s Yeast (*Saccharomyces cerevisiae*) as a Function of Different Sample Preparation Procedures

**DOI:** 10.3390/metabo10080301

**Published:** 2020-07-23

**Authors:** Lena Gmelch, Daniela Wirtz, Michael Witting, Nadine Weber, Lisa Striegel, Philippe Schmitt-Kopplin, Michael Rychlik

**Affiliations:** 1 Chair of Analytical Food Chemistry, Technical University of Munich, 85354 Freising-Weihenstephan, Germany; lena.gmelch@tum.de (L.G.); ga65way@mytum.de (D.W.); michael.witting@helmholtz-muenchen.de (M.W.); nadine1.weber@tum.de (N.W.); lisa.striegel@tum.de (L.S.); 2 Research Unit BioGeoChemistry, Helmholtz Zentrum Munich, 85764 Neuherberg, Germany

**Keywords:** folate metabolism, folate polyglutamates, baker’s yeast, solid phase extraction, *s*-pyrazino-triazine (MeFox)

## Abstract

Folates are a group of B_9_ vitamins playing an important role in many metabolic processes such as methylation reactions, nucleotide synthesis or oxidation and reduction processes. However, humans are not able to synthesize folates de novo and thus rely on external sources thereof. Baker’s yeast (*Saccharomyces cerevisiae*) has been shown to produce high amounts of this vitamin but extensive identification of its folate metabolism is still lacking. Therefore, we optimized and compared different sample preparation and purification procedures applying solid phase extraction (SPE). Strong anion exchange (SAX), C18 and hydrophilic–lipophilic-balanced (HLB) materials were tested for their applicability in future metabolomics studies. SAX turned out to be the preferred material for the quantitative purification of folates. Qualification of several folate vitamers was achieved by ultra-high pressure liquid chromatography quadrupole time of flight mass spectrometry (UHPLC-Q-ToF-MS) measurements and quantification was performed by liquid chromatography tandem mass spectrometry (LC-MS/MS) applying stable isotope dilution assays (SIDAs). The oxidation product *s*-pyrazino-triazine (MeFox) was included into the SIDA method for total folate determination and validation. Applying the best protocol (SAX) in regard to folate recovery, we analyzed 32 different vitamers in different polyglutamate states up to nonaglutamates, of which we could further identify 26 vitamers based on tandem-MS (MS^2^) spectra. Total folate quantification revealed differences in formyl folate contents depending on the cartridge chemistry used for purification. These are supposedly a result of interconversion reactions occurring during sample preparation due to variation in pH adjustments for the different purification protocols. The occurrence of interconversion and oxidation reactions should be taken into consideration in sample preparation procedures for metabolomics analyses with a focus on folates.

## 1. Introduction

Metabolomics studies usually are performed to obtain a deeper insight into the metabolism of the organism investigated [1,2,3]. This information is then used to elucidate metabolic pathways [4,5], or to determine biomarkers for diseases [6,7,8], or influences of outer circumstances on metabolism [9,10,11,12,13]. To avoid artifacts generated by degradation reactions, typical sample preparation procedures are rather gentle [3,14,15,16]. Furthermore, ice-cold temperatures throughout the sample preparation procedure or boiling in aqueous ethanol solutions are applied to stop metabolic processes by denaturing enzymes [1,3,17,18]. To date, mostly metabolites with the highest abundance are typically investigated [3,18,19], but minor compounds such as vitamins are not analyzed in detail. However, vitamins such as folates are known to be inalienable for a proper functioning metabolism [20,21,22,23].

Folates are a group of vitamers referring to the term vitamin B_9_, which is commonly known as folic acid. Folate vitamers are known to be involved in C_1_ metabolism by providing C_1_-units for the purine and pyrimidine synthesis as well as the synthesis of thymidylate [24,25,26,27]. Furthermore, folates are involved in the regulation of several neurotransmitters and the re-methylation of homocysteine to methionine [20,28,29,30]. Thus, intracellular accumulation of homocysteine is prevented. Increased homocysteine levels are linked to neurogenerative diseases such as Alzheimer’s disease [31,32]. Furthermore, increased risk for cardiovascular diseases [33,34,35], or prevalence of certain types of cancer [29,30] are reported to be linked to an insufficient folate supply. Most severe is the incident of neural tube defects in newborns after a maternal lack in folates during pregnancy [27,36]. Humans, however, are not able to synthesize folates de novo and thus are dependent on an adequate external supply with folates [37]. Deficiencies are usually overcome by supplementation or fortification with folic acid [38,39,40,41]. However, in contrast to synthetic supplements, natural folates have not been linked to increased risk of colorectal cancer [29,38,42]. Several biofortification approaches have already shown the potential of promoting folate metabolism by the metabolic engineering and upregulation of enzymes involved in folate metabolism [43,44,45,46,47,48]. However, more information about folate metabolism is needed for targeted upregulation of several folate vitamers and to increase the stability thereof.

**Figure 1:**
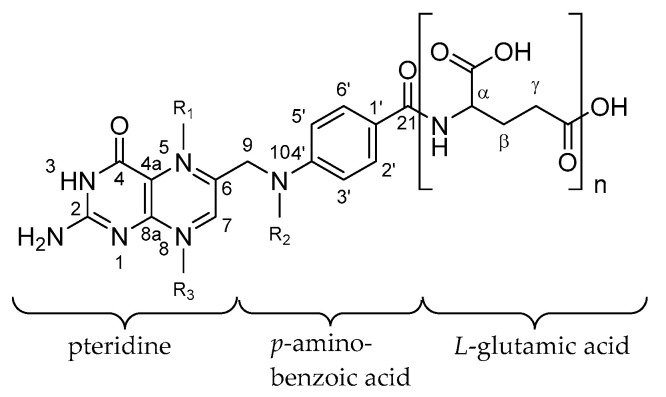
Molecular structure of main folate vitamers known so far.

Figure 1 and Table 1 give an overview of the core structure of folates: pteridine is linked with *p*-amino-benzoic acid and one to fourteen γ-linked L-glutamate moieties. Differences in the vitamers refer to the different oxidation states of the pteridine ring, different C_1_-substitution at positions C^5^ and C^10^ and different lengths of the polyglutamate tail. Due to those variations, more than 150 different vitamers are expected to exist in vivo [20,49,50].

**Figure 2:**
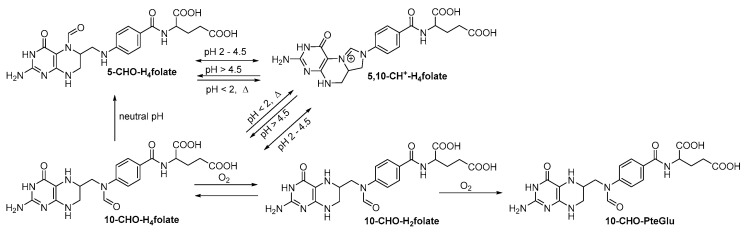
Interconversion reactions of formyltetrahydrofolate vitamers depending on external conditions.

**Figure 3:**
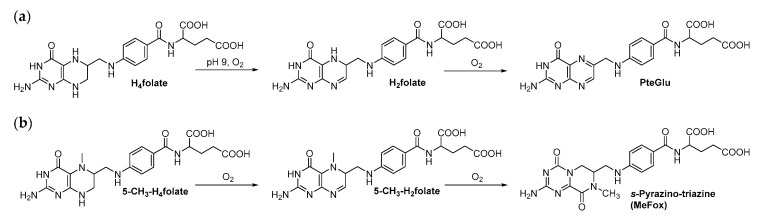
Oxidation reactions of selected folate vitamers: (**a**) oxidation reaction of H_4_folate, (**b**) oxidation reaction of 5-CH_3_-H_4_folate.

Folates are sensitive towards light, oxygen, increased temperatures or harsh pH conditions, which makes their analysis rather challenging [51,52,53,54]. To prevent degradation during sample preparation, the use of antioxidants such as ascorbic acid and thiols is inevitable [55,56,57]. Furthermore, extraction usually is performed under subdued light to avoid induction of oxidation reactions. Figure 2 summarizes the interconversion reactions, which are reported for formyl folates [54,58,59]. In brief, under physiological conditions, an equilibrium exists between 5-CHO-H_4_folate, 5,10-CH^+^-H_4_folate and 10-CHO-H_4_folate. A low pH will shift this equilibrium towards the 5,10-CH^+^-H_4_folate form, whereas neutral and alkaline conditions increase 10-CHO-H_4_folate formation. This vitamer can then be further oxidized into 10-CHO-H_2_folate and 10-CHO-PteGlu. Further oxidation reactions are shown in Figure 3. H_4_folate is oxidized to PteGlu and 5-CH_3_-H_4_folate is oxidized into the degradation product *s*-pyrazino-triazine, commonly known as MeFox [60,61,62].

So far, little is known about the folate metabolism at the polyglutamate level in general. Almost the entire research focuses on the respective monoglutamates after enzymatic deconjugation of the polyglutamate vitamers [20,49,63]. Only a few studies focused on the polyglutamate distribution of mammalian tissue [64,65], bacteria [65] and raw vegetables [64,66,67,68]. Yeast, however, has not been analyzed in terms of polyglutamate vitamers which typically account for the major occurring form in vivo. Polyglutamates have a decreasing influence on the bioavailability of folates in food due to reduced absorption rates thereof. [69] Thus, a simple quantification of total folate contents in yeasts after enzymatic deconjugation might not be sufficient enough and lead to inaccurate recommendations for the daily intake of this type of food. Therefore, there is a clear need to gain deeper insight into the folate metabolism in baker’s yeast (*Saccharomyces cerevisiae*), with special focus on polyglutamate metabolites.

The goal of this study was to compare different extraction and analysis methods for the profiling of folate metabolites in baker’s yeast. By doing this, we wanted to identify reasons for neglecting vitamins in metabolomics studies undertaken so far. We aimed to determine the best method for complete folate extraction to be applied for future studies of folate metabolites in yeast samples. Yeast was chosen as a model organism due to its relatively high folate contents of around 4000 µg/100 g [70,71,72] and good applicability for further metabolic engineering studies and/or biofortification programs.

Typical sample preparation protocols for the analysis of folates include extraction in a boiling water bath for the inactivation of enzymes and liberation of intracellularly bound folates [63,73]. Further purification and concentration usually are achieved by solid phase extraction (SPE). Solid phase extraction of folate extracts mostly is performed using strong anion exchange materials based on polymerically bonded quaternary amine [74,75,76,77]. Alternative materials such as weak anion exchange materials [78], phenyl [74,79,80,81] or C18 [82,83,84]-modified materials are used less frequently. Few studies also reported the use of hydrophilic–lipophilic-balanced (HLB) materials consisting of *N*-vinylpyrrolidon-divinylbenzol copolymers offering application for a broad range of analytes [66,85]. We wanted to compare different protocols for their applicability to studies of folate metabolites. By doing so, we wanted to identify the most appropriate protocol for future studies.

## 2. Results

For analyzing the metabolite profile of yeast samples with a focus on folate metabolites, different purification procedures by SPE were established. To unravel differences in the applied purification procedures in more depth, we focused on the qualitative and quantitative analysis of metabolites belonging to the folate group. Qualification and quantification of folate metabolites demonstrated not only different coverage of vitamers but also shifts in the vitamer distribution depending on the cartridge material used for purification.

### 2.1. Optimization of Sample Preparation Procedures for the Investigation of Folate Metabolites by UHPLC-Q-ToF-MS

To study folate metabolism in yeast samples by UHPLC-Q-ToF-MS (ultra-high pressure liquid chromatography quadrupole time of flight mass spectrometry) measurements, we first tried to apply simple shotgun approaches, which usually are used in studies of the metabolite profile of yeasts [14,15,86]. Detailed information about the applied extraction procedures are provided in the Supplementary Section. None of the tested approaches did reveal any MS feature assignable to a known member of folate metabolism. We assumed the lack of detection being a result of a generally low extraction yield applying extraction in solvent mixtures. Additionally, high matrix effects in the sample extracts leading to ion suppression in the ion source were suspected.

Therefore, we decided to include additional purification and concentration steps using solid phase extraction. Furthermore, the extraction procedures usually applied for the quantification of folate vitamers were adjusted to the needs of the present study. In a preliminary study design, we tested several cartridges (SAX, phenyl, amino propyl, C18, HLB and anion mixed mode cartridges; tested materials are listed in Supplementary Table S1), of which we selected the most promising candidates and optimized their purification protocols. Details about the pre-study and the optimization of the selected cartridge materials can be found in the Supplementary Section. Supplementary Table S2 gives details about the instrument settings used for analysis. In the end, we came up with the following four different procedures shown in Table 2: SAX applying an elution with an elution buffer containing 0.1 M sodium acetate (NaAc) and 5% sodium chloride (stated as procedure “SAXa” hereafter), and an alternative elution with 20% acetonitrile containing 5% formic acid (stated as ‘SAXb’); HLB applying an elution with methanol; and C18 applying an elution with 20% acetonitrile containing 0.1% formic acid, respectively.

Adapting the sample preparation procedure accordingly, the detection of folate vitamers was possible by UHPLC-Q-ToF-MS. Furthermore, matrix effects could be reduced as depicted by Supplementary Figure S1. Differences between the purification protocols such as reduced H_4_folate detection or increased detection of 5,10-CH^+^-H_4_folate (shown in Supplementary Figure S2) were observed for the alternative cartridge materials which we wanted to investigate further.

### 2.2. Qualitative Profiling of Folate Metabolites by UHPLC-Q-ToF-MS

**Figure 4:**
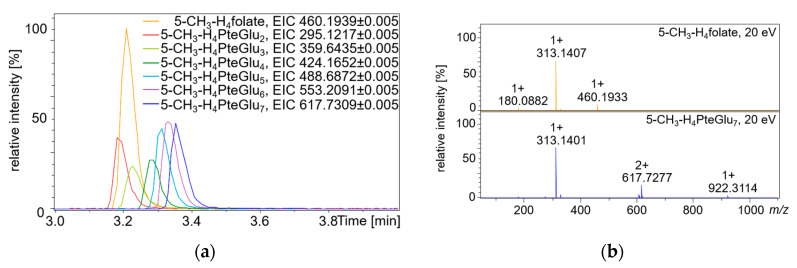
5-CH_3_H_4_folat standard and synthesized folate standards 5-CH_3_-H_4_PteGlu_2–7_ measured on the UHPLC-Q-ToF-MS after chromatographic separation on a Restek Raptor^TM^ ARC-18 column (1.8 µm, 100 × 2.1 mm): (**a**) extracted ion chromatograms (EIC) of the folate standards; (**b**) tandem MS/MS spectra of 5-CH_3_-H_4_folate and 5-CH_3_-H_4_-PteGlu_7_ at CE = 20 eV.

Running the commercially available folate standards and synthesized 5-CH_3_-H_4_folate polyglutamate standards under the chromatographic conditions used for this study, we received information such as retention behavior as well as fragmentation patterns (obtained from tandem MS/MS spectra, hereafter stated as MS^2^ spectra). As expected, we observed slightly longer retention on the chromatographic column with prolongation of the polyglutamate tail, as shown in Figure 4a. Fragmentation patterns, however, were similar between different polyglutamate vitamers of the measured polyglutamate standards 5-CH_3_-H_4_PteGlu_2–7_. The bonds of the polyglutamate tail turned out to be easily broken by moderate collision energies above 15 eV resulting in a neutral loss of polyglutamate moieties and retaining the monoglutamate core structure (comparative MS^2^ spectra of 5-CH_3_-H_4_folate and 5-CH_3_-H_4_PteGlu_7_ shown in Figure 4b). To confirm this observation, we synthesized the oxidation product MeFox-Glu_7_ and compared the retention behavior and fragmentation patterns of the heptaglutamate and the monoglutamate form, giving the same results (visualized in Supplementary Figure S3). As none of the polyglutamate standards are commercially available, we assumed their identification once the retention time (with addition of the shift due to prolongation of the glutamate tail) and MS^2^ spectra were in accordance with the respective monoglutamate standard.

**Figure 5:**
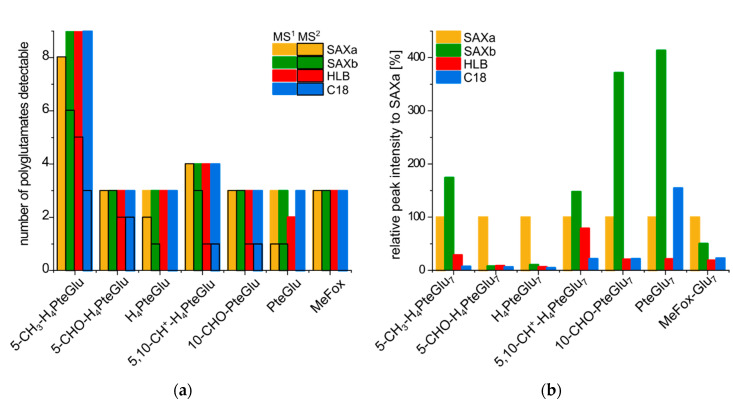
Qualitative analysis of the folate vitamers detectable in baker’s yeast samples purified by different solid phase extraction materials and measured with UHPLC-Q-ToF-MS with purification using SAXa: strong anion exchange (SAX) and elution with elution buffer; SAXb: SAX and elution with 20% acetonitrile and 5% formic acid; HLB: hydrophilic–lipophilic-balanced; C18: (**a**) number of detectable vitamers based on different vitamer groups at the MS^1^ level (columns) and MS^2^ level (black borders); (**b**) relative peak intensity for different heptaglutamates after normalization to SAXa.

Thus, we could focus on features eluting in the respective retention time windows referring to folates ± 10 s and reduce the obtained data matrix of MS^1^ features based on this knowledge. Comparing the obtained MS^1^ features belonging to folates with dependence on the solid phase extraction material (depicted by different colors in Figure 5a), we could not observe any bigger differences within each group of vitamers. Each of the purification procedures yielded a similar polyglutamate distribution in total. Solely the HLB material retained only two instead of three different pteroylglutamic acid polyglutamates (PteGlu_n_), whereas the SAX material in combination with the elution buffer (SAXa) bound only eight instead of nine different 5-methyl-tetrahydrofolate polyglutamates (5-CH_3_-H_4_-PteGlu_n_). However, the peak intensities of the features varied considerably. Figure 5b gives an overview of the relative peak intensity of the different vitamer groups after normalization to the standard procedure SAXa, here shown for the heptaglutamates. As can be observed, the SAX material generally provided the highest peak intensities and thus the best coverage of folate metabolites. The higher intensities for the alternative procedure SAXb for 10-CHO-PteGlu and PteGlu might be artifacts due to underlying oxidation reactions, which will be addressed in the subsequent sections in detail.

To further compare the different SPE procedures applied, we calculated the distribution of polyglutamates as a percentage of the total sum of intensity within each vitamer and SPE cartridge. By doing so, we wanted to check whether a discrimination of polyglutamates with dependence on the vitamer group and the SPE material had to be taken into account. Comparing the resulting of the graphs shown in Supplementary Figure S4a–g, no decisive variation can be observed for several folate vitamers across different SPE chemistries except for H_4_folates. In general, hexa-, hepta- and octaglutamates occurred as the main polyglutamate form for each of the vitamers identified. Supplementary Table S3 lists a detailed comparison of the percentage distribution of 5-CH_3_-H_4_PteGlu_1–9_, showing no further discrimination between the SPE cartridges used for purification.

Measurements in data-dependent acquisition (DDA) mode (details listed in Supplementary Table S4) enabled further investigation and identification of the folate MS^1^ features. Due to the introduced preference masses, MS^2^ spectra could also be obtained for folate features with less intense abundance (depicted by black borders in Figure 5a). Here, clear distinctions between cartridge materials could be drawn. For the SAX cartridges, almost each of the obtained folate MS^1^ features could additionally be confirmed at the MS^2^ level (81 and 72% for SAXa and SAXb, respectively). Yet sacrifices had to be made for the more general HLB and C18 material with only 28 and 22% of all obtained folate MS^1^ features being verified at the MS^2^ level, respectively. Only the application of SAX material could convincingly further verify the many peaks with a huge number of vitamers so far not reported in yeast as polyglutamates up to nonaglutamates. A summary of all detected MS^1^ folate features and their identification by MS^2^ spectra is listed in Supplementary Table S5. Moreover, MS^2^ spectra are provided in the Supplementary Materials in .mb file format.

### 2.3. Quantitative Analysis of the Total Folate Content after Enzymatic Deconjugation and Purification with Different SPE Materials

Quantification of the total folate contents was investigated after enzymatic deconjugation into the respective monoglutamates. Stable isotope dilution assays (SIDA) were applied to each of the tested purification procedures in triplicate (quadruples in the case of procedure SAXa). The use of isotopically labeled standards enables compensation for a loss of analyte during sample preparation or ion suppression in LC-MS/MS analysis as analytes and standards are assumed to be affected in the same way. Furthermore, the so-called carrier effect might improve the quantitation of trace amounts of analytes [87]. Spiking amounts of the different internal standards (ISTD) had to be adjusted individually to give peak ratios of A(A)/A(ISTD) in the linear range of the calibration curves. To investigate oxidation reactions during sample preparation in detail, the oxidation product MeFox (*s*-pyrazino-triazine) was included into the quantitative method and validated (details of the validation are shown in the Supplementary Materials, validation results are listed in Supplementary Table S6).

**Figure 6:**
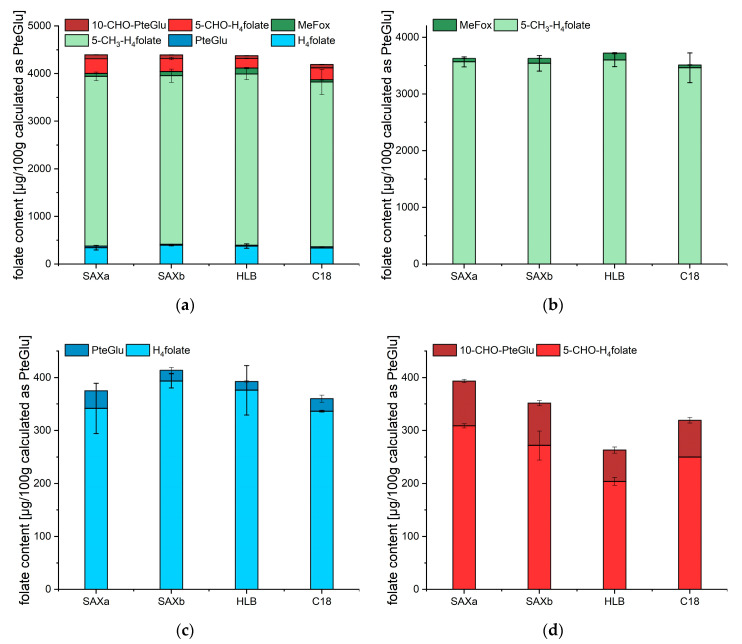
Quantitative analysis of the folate vitamers in baker’s yeast samples purified by different solid phase extraction materials and measured with UHPLC-LC-MS/MS applying stable isotope dilution analysis (SIDA) with purification using SAXa: strong anion exchange (SAX) and elution with elution buffer; SAXb: SAX and elution with 20% acetonitrile and 5% formic acid; HLB: hydrophilic–lipophilic-balanced; C18. (**a**) Total folate analysis for different vitamers, (**b**) total folate analysis for 5-CH_3_-H_4_folate and MeFox, (**c**) total folate analysis for H_4_folate and PteGlu and (**d**) total folate analysis for 5-CHO-H_4_folate and 10-CHO-PteGlu.

Figure 6 gives an overview of the obtained results for the sum of all analyzed vitamers (Figure 6a) as well as for single vitamers (Figure 6b–d). The overall sum of the vitamers was in the same range between 4188 ± 206 (C18) and 4396 ± 89 µg/100 g (SAXa) for all four procedures. 5-CH_3_-H_4_folate determined after enzymatic deconjugation was constant for the different SPE materials, ranging between 3462 ± 263 for the C18 procedure and 3598 ± 118 µg/100 g for the HLB procedure, as shown in Figure 6b. The oxidation product MeFox did also not strongly alter these results and ranged between 46.9 ± 8.1 for the C18 procedure and 123.1 ± 15.5 µg/100 g for the HLB procedure, respectively.

The more labile vitamer H_4_folate (visualized in Figure 6c) showed higher variation between 336.2 ± 1.4 for the C18 procedure and 393.6 ± 13.5 µg/100 g for the SAXb procedure. These variations could not fully be compensated for by the oxidation product PteGlu, which ranged between 16.7 ± 1.2 for the HLB procedure and 33.0 ± 3.5 µg/100 g for the SAXa procedure.

5-CHO-H_4_folate and 10-CHO-PteGlu (shown in Figure 6d) revealed the same decreasing tendency. Highest values were obtained by the SAXa procedure (308.8 ± 4.06 for 5-CHO-H_4_folate and 84.7 ± 3.3 µg/100 µg for 10-CHO-PteGlu) and lowest values were obtained by the HLB procedure (203.9 ± 7.2 for 5-CHO-H_4_folate and 59.0 ± 5.6 µg/100 g for 10-CHO-PteGlu).

## 3. Discussion

It is commonly accepted that sample preparation is a crucial step for metabolomics analyses [88,89,90,91]. Therefore, the appropriate sample extraction procedure had to be chosen prior to analysis. Many studies have revealed the need for complete extraction, on the one hand, and prevention of extensive degradation, on the other hand. Degradation can be caused by enzymatic activity leading to interconversion reactions, and chemical and/or thermal influences [89,91]. Quenching to stop metabolic activity usually is achieved by the addition of ice-cold methanol. After centrifugation, this method additionally enables a separate analysis of intracellular and extracellular metabolites for the residue and the supernatant, respectively [90,91]. Since our primary goal was folate metabolites, which are known to occur intracellularly [71,72], we focused on the optimization of extraction procedures for intracellular metabolites.

Comparison of different metabolomics studies of baker’s yeast reveals contradictory results in terms of the ideal extraction technique. [88,89,92] Thus, we tested different combinations of organic solvents usually applied in metabolomics analyses [14,15,86], with the variation in the extraction temperature between 0, 20 and 80 °C. None of the applied methods led to the effective extraction of folate vitamers presumably due to sample concentrations in the µM physiological range and insufficient folate extraction. In comparison with improved folate solubility in buffer solutions such as phosphate buffer or MES (2-(*N*-morpholino)-ethanesulfonic acid) buffer followed by a heating step commonly used in folate analysis [20,49], extraction in solvent mixtures seemed not to be an adequate sample preparation protocol. Therefore, we decided to optimize the extraction procedure accordingly, to increase the initial sample weight and extraction volume, and additionally to introduce a concentration step by solid phase extraction. By doing so, we furthermore expected to achieve less ion suppression in mass spectrometric analyses due to reduced matrix effects in relation to higher analyte concentrations.

The metabolite composition of the final sample extract is highly dependent on the SPE material used for purification. Several studies have shown that the application of solid phase extraction can help to increase metabolome coverage by improving sensitivity up to a factor of 75 [93,94]. However, the applied elution with 1% formic acid in the case of anionic protocols and 0.1% formic acid in 3% acetonitrile or methanol for RP protocols might not be efficient enough. Only 5% formic acid concentrations for SAX materials and 20% acetonitrile acidified with 0.1% formic acid for C18 materials achieved convincing extraction of folate vitamers

In our studies, successful optimization of each SPE procedure for efficient folate extraction could be demonstrated by a wide coverage of the folate metabolome at the MS^1^ level. Many vitamers so far not reported in yeast [71,72,95] could be tentatively identified up to nonaglutamates. Each of the tested purification procedures achieved a similar coverage, yet peak intensities varied substantially. On average, the SAXb procedure obtained the highest peak intensities due to the further concentration of the SPE eluates. However, oxidation reactions could be observed by decreased intensities of the labile H_4_PteGlu_n_ vitamers and increased intensities of the oxidation products 10-CHO-PteGlu_n_ and PteGlu_n_. These oxidation reactions might be caused by the acidic conditions of the final sample extract and concentration effects thereof during evaporation. Lower peak intensities for 5-CHO-H_4_PteGlu_n_ possibly are another result of this concentration effect. We hypothesize that this observation can be traced back to underlying oxidation and interconversion reactions. First, neutral conditions in combination with the use of antioxidative agents in the elution buffer (procedure SAXa) promote a shift of all formyl vitamers into the 10-CHO-H_4_PteGlu_n_ form, which we then cannot separate from its isomer 5-CHO-H_4_PteGlu_n_. Different previous studies with monoglutamate standards have shown these interconversions [58,59]. Second, the acidic conditions in procedure SAXb favor the interconversion of all formyl vitamers into the respective 5,10-CH^+^-H_4_PteGlu_n_ forms, which are more stable under those conditions as shown by Brouwer et al. [54]. The same trends could be observed for the other two procedures HLB and C18 at both the qualitative polyglutamate level and the quantitative monoglutamate level after enzymatic deconjugation. Both SPE protocols need an adjustment of the sample pH to pH 2 prior to solid phase extraction. Additionally, the elution solution of the C18 procedure is characterized by acidic conditions (0.1% formic acid). Overcoming the problem of acidification by applying alkaline SPE protocols (such as amino propyl cartridge) did not result in sufficient recovery as shown in the Supplementary Materials and therefore cannot provide an applicable alternative.

On average, HLB and C18 cartridges only reached about 26 and 34% peak intensity for folates compared with the SAXa procedure, respectively. On the one hand, this highlights the specificity of the SAX material towards folates while, on the other hand, it demonstrates the broader metabolite spectrum which can be obtained by those more general SPE materials. As a result of the decreased peak intensities for the procedures HLB and C18, further identification at the MS^2^ level could not be achieved to the same extent compared with SAXa and SAXb, respectively. While that for the procedures SAXa and SAXb, 70 to 80% of the MS^1^ features could be verified by the corresponding MS^2^ spectra, this was only the case for 20–30% of the HLB and C18 eluates.

In contrast to qualitative polyglutamate determinations, the use of the ideal internal standard in the case of the total folate determination after enzymatic deconjugation into the respective monoglutamate vitamers should counterbalance the different behavior and recovery of folate standards on the SPE materials. This can clearly be seen by steady results for the sum of all folate vitamers analyzed. Further, values for 5-CH_3_-H_4_folate were quite constant as well as the oxidation product MeFox, highlighting the relative stability of this vitamer. In contrast to this, the more labile vitamer H_4_folate showed changes between the different purification procedures which was also observable for the percentage distribution of the polyglutamates analyzed by UHPLC-Q-ToF-MS. Those variations were also not fully reflected by its respective oxidation product PteGlu. This suggests further oxidation into other degradation products, which cannot be covered by the method applied for quantification. 5-CHO-H_4_folate and 10-CHO-PteGlu contents followed the same order SAXa > SAXb > C18 > HLB. Accordingly, those two analytes seem to behave similarly, showing the same dependency on the purification procedure applied. This underlines the connection between those two vitamers. As shown by several studies, 10-CHO-PteGlu evolves as the oxidation product of 10-CHO-H_4_folate (see Figure 2) [59,96]. Since both vitamers show the same trends in terms of folate contents across different SPE procedures, it can be assumed that oxidation reactions only have a minor influence on the vitamer composition compared with interconversion reactions. Jastrevoba et al. investigated the dependency of 5-CHO-H_4_folate during sample preparation procedures. They could demonstrate that interconversion reactions will preferably lead to the formation of 5-CHO-H_4_folate at a pH above 4.8. Thus, all formyl vitamers present in the sample will be interconverted into 5-CHO-H_4_folate during extraction using MES buffer adjusted to pH 5. As a result, the determination by the SAXa procedure most likely gives the sum of all formyl vitamers quantitated as 5-CHO-H_4_folate. Smaller values for the SAXb procedure, however, demonstrate partial interconversion into 5,10-CH^+^-H_4_folate since elution from the SAX cartridges occurs under acidic conditions. Procedure C18 is characterized by even lower values for 5-CHO-H_4_folate compared with the SAXb procedure. This observation is also in accordance with the conditions during sample preparation where an acidic milieu already had been adjusted prior to solid phase extraction. Hence, acidic conditions are present for about 2.5 to 3 h (procedure C18) throughout the whole SPE procedure compared with about 30 min (SAXb procedure) until complete evaporation of the eluate. Consequently, the shift towards 5,10-CH^+^-H_4_folate, which has been proven to be stable in pH < 2 will be even more drastic [59]. The HLB procedure also had an acidic milieu for 2.5 to 3 h, resulting in lower values for 5-CHO-H_4_folate compared with the SAXa procedure. It can be assumed that both 5-CHO-H_4_folate and 5,10-CH^+^-H_4_folate are less stable in the fully organic solvent (MeOH) used for elution in the HLB procedure compared with the acidic aqueous acetonitrile solution (20% acetonitrile with 0.1% formic acid). As the oxidation product 10-CHO-PteGlu also shows the smallest values for the HLB procedure, we assume that further oxidation reactions occur, leading to degradation products, which we cannot determine with our quantitative method.

Quantification of the total folate content after summation of the individual folate vitamers analyzed gave values between 4188 ± 206 and 4396 ± 89 µg/100 g for all four procedures. These findings are in accordance with previously reported folate contents in baker’s yeast, typically ranging between 1000 and 4000 µg/100 g [70,71,72,97]. Only the specific cultivation of yeast strains resulted in total folate contents between 4000 and 14,500 µg/100 g [72,98]. Each of the studies identified 5-CH_3_-H_4_folate and H_4_folate as the main vitamers. Amounts of 5-CH_3_-H_4_folate ranged between 65 and 77% of the total folate content, which is somewhat lower than the 80.5–82.7% observed with our studies. H_4_folate contents accounted for 7.8–9.0% of the total folate content in our investigations. Those findings are lower than the percentage of 20 to 25% analyzed previously [70,71]. However, the latter data were obtained from HPLC-UV and HPLC-fluorescence, respectively. Thus, lacking in sensitivity, they could not quantify any other folate vitamers. With our method, however, we were able to furthermore quantify 5-CHO-H_4_folate (4.7–7.0%), 10-CHO-PteGlu (1.3–1.9%), folic acid (0.8–1.8%) and the oxidation product MeFox (1.2–2.8%). Besides that, we could clearly determine 5-CH_3_-H_4_folate to be the main occurring vitamer.

To the best of our knowledge, this is the first study of the polyglutamate pattern with regard to different vitamers in baker’s yeast. So far, only Schertel et al. found 97% of the folate vitamers containing three or more glutamate moieties in the polyglutamate chain. Determination of different species and the exact lengths, however, was not possible with the latter method. Ndaw et al. analyzed the general polyglutamate pattern after conversion of all polyglutamates into the corresponding 5-CH_3_-H_4_PteGlu_n_ vitamer. The latter authors found the hexa-, hepta- and octaglutamate forms to be the only occurring polyglutamates in baker’s yeast. However, they could not distinguish between the different vitamer forms. Our studies revealed the hexa-, hepta- and octaglutamate forms to be the predominant forms in baker’s yeast for each of the detected vitamers (5-CH_3_-H_4_folate, 5-CHO-H_4_folate, H_4_folate, 5,10-CH^+^-H_4_folate, 10-CHO-PteGlu, PteGlu and the oxidation product MeFox). The longest glutamate chains were nonaglutamates for the vitamers 5-CH_3_-H_4_folate, 5-CHO-H_4_folate, H_4_folate and 5,10-CH^+^-H_4_folate.

## 4. Materials and Methods

### 4.1. Chemicals and Materials

Unless otherwise stated, chemicals were of analytical grade. Acetonitrile, methanol, ethanol, isopropanol and water were purchased from VWR (Darmstadt, Germany); 2-(*N*-morpholino)-ethanesulfonic acid (MES) hydrate, ascorbic acid, formic acid (>95%), sodium acetate trihydrate, hydrogen peroxide, dithiothreitol (DTT), sodium thiosulfate, sodium iodide, hydrogen peroxide, α-cellulose, oleic acid, l-isoleucine, l-leucine, l-lysine, l-threonine and l-valine from Sigma-Aldrich (Steinheim, Germany); disodium hydrogen diphosphate, potassium dihydrogen phosphate and dipotassium hydrogen phosphate from Merck (Darmstadt, Germany); sodium chloride and sodium hydroxide from VWR (Darmstadt, Germany); and rat serum and chicken pancreas containing γ-glutamyl hydrolase (EC 3.4.19.9) from Biozol (Eching, Germany) and Difco (Sparks, MD, USA), respectively. Acetonitrile (hypergrade from LC-MS) for the high-resolution (HR) mass spectrometry (MS) measurements was purchased from Merck (Darmstadt, Germany) and MilliQH_2_O was derived from Milli-Q Integral Water Purification System (Billerica, MA, USA).

Folate standards (5-CH_3_-H_4_folate, 5-CHO-H_4_folate, 5,10-CH^+^-H_4_folate, H_4_folate, 10-CHO-PteGlu, PteGlu and PteGlu_2–7_) and labeled internal standards ([^13^C_5_]-5-CH_3_-H_4_folate, [^13^C_5_]-5-CHO-H_4_folate), [^13^C_5_]-H_4_folate and [^13^C_5_]-PteGlu) were purchased form Schircks Laboratories (Jona, Switzerland), whereas MeFox was obtained from Merck & Cie (Schaffhausen, Switzerland). Strata strong anion exchange (SAX) cartridges (quaternary amine, 500 mg, 3 mL, and 1 g, 6 mL) and Strata C18-T cartridges (500 mg, 3 mL) were purchased from Phenomenex (Aschaffenburg, Germany); Chromabond hydrophilic-lipophilic-balanced (HLB) cartridges (60 µm, 500 mg, 3 mL) were obtained from Macherey-Nagel (Düren, Germany).

### 4.2. Solutions for Sample Extraction and Solid Phase Extraction Procedures

The extraction buffer for the total folate determination was prepared by adjusting an aqueous solution of 100 mmol/L MES hydrate, 10 g/L ascorbic acid and 1 g/L DTT with 5 M NaOH to pH 5.0. For the phosphate buffer, an aqueous solution of disodium hydrogen phosphate (100 mmol/L) was adjusted to pH 7.0 by addition of an aqueous solution of potassium dihydrogen phosphate buffer (100 mmol/L). Extraction buffer for the polyglutamate determination consisted of 100 mmol/L dipotassium hydrogen phosphate and 10 g/L ascorbic acid aqueous solution with 1 g/L DTT. The pH was adjusted to pH 5.0 with 5 M NaOH. Equilibration buffer for the SAX cartridges was prepared by addition of 0.1 g/L DTT to diluted phosphate buffer (10 mmol/L). Equilibration buffer for the C18 and HLB cartridges was additionally adjusted to pH 2.0 with formic acid. Elution buffer for the SAX cartridges consisted of a mixture of aqueous sodium acetate (100 mmol/L) containing 10 g/L ascorbic acid and 1 g/L DTT. Further, the elution buffer contained an aqueous solution of 5% sodium chloride.

Chicken pancreas solution for the pteroylpolyglutamate deconjugation was prepared by stirring 1 g/L chicken pancreas in phosphate buffer (100 mmol/L) containing 1 g/L ascorbic acid which had been adjusted for pH 7.0 by 5 M NaOH. Rat serum was used without any further dilution.

### 4.3. Sample Collection

Baker’s yeast was bought in a local supermarket in Germany. Samples were frozen, lyophilized and homogenized prior to analysis. The dried samples were kept at −20 °C until the day of analysis.

### 4.4. Sample Extraction Procedure for the Analysis of the Total Folate Content

Sample preparation for the total folate determination was as reported elsewhere [99] with slight adjustments to the sample purification. For purification by SAX cartridges, 5 mL of methanol was added prior to centrifugation. For purification by C18 or HLB cartridges, 5 mL of extraction buffer was added prior to centrifugation at 4000× *g* at 4 °C for 20 min. C18 and HLB sample extracts were adjusted to pH 2.0 by addition of formic acid and centrifuged at 4000× *g* at 4 °C for another 20 min.

### 4.5. Sample Extraction Procedure for the Analysis of the Pteroylpolyglutamates

Procedures for the extraction of the pteroylpolyglutamates from yeast samples were as for the total folate determination with some minor modifications. In brief, 1 g of dried yeast sample was equilibrated with 10 mL extraction buffer (phosphate buffer) for 15 min. Subsequently, samples were vortex-mixed for 30 s followed by cooling on ice for another 30 s with a total time of 15 min to enable a complete destruction of cell membranes. For further liberation of protein-bound folates, samples were heated at 100 °C for 10 min and immediately cooled on ice afterwards. After addition of 5 mL acetonitrile (for SAX cartridges) or 5 mL extraction buffer (for C18 and HLB cartridges), the extracts were centrifuged at 4000× *g* at 4 °C for 20 min. For purification by C18 or HLB cartridges, the pH was adjusted to pH 2.0 by addition of formic acid prior to a second centrifugation step at 4000× *g* at 4 °C for 20 min.

### 4.6. SPE-Procedure for Sample Extracts with SAX Cartridges (Procedures SAXa and SAXb)

Different sizes of cartridges were used depending on the analysis applied. A bed volume of 500 mg (3 mL) was used for total folate determinations while a bed volume of 1 g (6 mL) was used for profiling analyses. Cartridges were successfully activated by the addition of 2 volumes of equilibration buffer and 2 volumes of methanol prior to application of the sample extract. For washing, 2 volumes of equilibration buffer were applied (2 volumes of water adjusted to pH 5.0 were used for UHPLC-Q-ToF-MS measurements, respectively). For the total folate determination, folates were eluted with 2 mL of elution buffer. For the polyglutamate profiling, 6 mL of elution buffer was used. Sample extracts were stored at −20 °C until analysis.

For the alternative purification procedure with SAX cartridges (stated as procedure SAXb), cartridge size, conditioning, sample loading and washing steps were as described above. For the extraction, however, a solution consisting of 20% acetonitrile and 5% formic acid was used. Volumes for elution depended on the applied analysis: total folate extracts were eluted with 2 mL, whereas extracts for the polyglutamate profiling were eluted with 6 mL. Sample extracts were evaporated under nitrogen gas flow in aliquots of 1 mL and stored at −20 °C. Prior to analysis, samples were reconstituted in 200 µL of elution buffer (for analysis by LC-MS/MS) or 200 µL 1% formic acid (for analysis by UHPLC-Q-ToF-MS).

### 4.7. SPE-Procedure for Sample Extracts with HLB Cartridges

HLB cartridges (500 mg, 3 mL) were successfully activated by 2 volumes of equilibration buffer adjusted to pH 2.0. After application of the sample extract, 6 mL of water adjusted to pH 2.0 was applied for washing. Folates were eluted with 2 mL for the total folate determination. For the polyglutamate profiling, 6 mL methanol was used. Sample extracts were evaporated under nitrogen gas flow in aliquots of 1 mL and stored at −20 °C until analysis. Prior to analysis, samples were reconstituted in 200 µL of elution buffer (for analysis by LC-MS/MS) or 200 µL 1% formic acid (for analysis by UHPLC-Q-ToF-MS).

### 4.8. SPE-Procedure for Sample Extracts with C18 Cartridges

C18 cartridges (500 mg, 3 mL) were successfully activated by 2 volumes of equilibration buffer adjusted to pH 2.0 prior to application of the sample extract. Cartridges were washed with 3 mL of water adjusted to pH 2.0 and 3 mL of 5% MeOH containing 0.1% formic acid. Folates were eluted with 2 mL of an elution solution consisting of 20% acetonitrile and 0.1% formic acid for the total folate determination. For the polyglutamate profiling, 6 mL of the same elution solution was used depending on the polyglutamates analyzed. Analogous to the HLB procedure, sample extracts were evaporated under nitrogen gas flow in aliquots of 1 mL and stored at −20 °C until analysis. Evaporated samples were reconstituted in 200 µL of elution buffer (for analysis by LC-MS/MS) or 200 µL of 1% formic acid (for analysis by UHPLC-Q-ToF-MS).

### 4.9. Determination of Folate Standard Concentrations by High Performance Liquid Chromatography Diode Array Detector (HPLC-DAD)

Analyte stock solutions were prepared by dissolving 10 mg of PteGlu in 100 mL MES buffer and 2 mg of 5-CH_3_-H_4_folate, H_4_folate, 5-CHO-H_4_folate, 10-CHO-PteGlu and MeFox in 10 mL MES buffer. Concentrations of analyte solutions were analyzed using the HPLC-DAD method published before [99].

### 4.10. Determination of the Total Folate Content by LC-MS/MS

Concentrations of the internal standard solutions used for spiking during sample extraction had to be determined for each extraction. Therefore, analyte stock solutions were diluted with MES buffer to a final concentration of 1 mg/mL and mixed with the internal standard solutions. This calibrator solution was measured together with the sample extracts.

Samples were measured after chromatographic separation on a C18-column (Raptor^TM^ ARC-18, 2.7 µm, 100 × 2.1 mm, Bad Homburg, Germany) in multi-reaction monitoring mode (MRM) as described elsewhere [99]. Details of the measured MRM transitions can be found in Supplementary Table S7.

### 4.11. Synthesis of 5-CH_3_-H_4_PteGlu_n_ and MeFox-PteGlu_7_

5-CH_3_-H_4_folate polyglutamates were synthesized by methylating the respective pteroylpolyglutamate PteGlu_2–7_ according to the procedure of Ndaw et al., as previously published [100]. 5-CH_3_-H_4_PteGlu_7_ was used for oxidation into the respective polyglutamate MeFox-PteGlu_7_ according to the procedure of Gapski et al. [62], with slight modifications. In brief, 500 µL of the 5-CH_3_-H_4_PteGlu stock solution (c = 6381 nmol/L in Tris-buffer) was incubated with 200 µL aqueous H_2_O_2_ (30%) and stirred for 2 h. Remaining hydrogen peroxide was quenched by adding 100 µL Na_2_S_2_O_3_ (1 M) and a spatula of NaI. The solution was diluted and directly injected into the LC-MS system.

### 4.12. Optimization of the Analysis of the Folate Metabolite Profile by UHPLC-Q-ToF-MS

Chromatographic separation for the analysis of the folate profile in yeast samples was performed using a Waters Acquity UHPLC System (Waters, Eschborn, Germany) coupled to a Bruker maXis^TM^ UHR-ToF-MS with an Apollo II ESI source (Bruker Daltonics, Bremen, Germany). Different UHPLC columns were tested to determine the best performing column. Furthermore, different gradients and different concentrations of formic acid in the mobile phase were tested. Best results were obtained using a modified C18 column (Restek Raptor^TM^, Bad Homburg, Germany, ARC-18, 1.8 µm, 100 × 2.1 mm) with water and acetonitrile containing 1% formic acid each serving as mobile phases A and B, respectively. The flow rate was set to 0.3 mL/min and the gradient for separation was as follows: 3.7 min pre-run time, 3% B; 0–1 min, 3% B; 1–2.25 min, 10% B; 2.25–3.5 min, 10% B; 3.5–6.5 min, 50% B; 6.5–7 min 50% B; 7–7.3 min 99.9% B;.3–9 min, 99.9% B; 9–9.3 min, 3% B. The column was maintained at 30 °C and 15 µL of sample was injected by full loop.

Data were collected in positive scan mode applying the data-dependent acquisition mode (DDA) with fragmentation of the three most abundant peaks per scan. To acquire fragmentation patterns for the less abundant folate vitamers, a preference list was set according to the expected folate vitamers. Details thereof can be found in Supplementary Table S4. The ESI parameters were as follows: nitrogen flow rate of 10 L/min, dry heater of 200 °C, nebulizer pressure of 2.0 bar and capillary voltage of 4500 V. Data were acquired with an acquisition rate of 5 Hz at a mass range from *m/z* 50 to *m/z* 1500. Calibration of the mass spectrometer was performed by injecting ESI-L Low Concentration Tuning Mix (Agilent, Santa Clara, CA, USA). Internal calibration of the obtained mass spectral data was achieved by injecting ESI-L Low Concentration Tuning Mix (1:4 diluted in acetonitrile) in the first 0.3 min of each LC-MS run.

### 4.13. Data Processing

Raw data obtained from measurements at the Q-ToF-MS were processed using the Genedata Expressionist for Mass Spectrometry 13.5 software (Genedata GmbH, Munich, Germany). Data transformation steps included chemical noise subtraction, intensity cutoff filter, calibration, retention time alignment, chromatographic peak picking, peak clustering, adduct detection and blank subtraction.

MS^1^ and MS^2^ spectra were further investigated in the R software version 3.6.1 (Boston, MA, USA) using the package MSnbase [101]. Identification of folate vitamers was confirmed by injecting several folate monoglutamate standards commercially available (5-CH_3_-H_4_folate, 5-CHO-H_4_folate, H_4_folate, 5,10-CH^+^-H_4_folate, 10-CHO-PteGlu, PteGlu, MeFox). Furthermore, the synthesized pteroylpolyglutamates 5-CH_3_-H_4_PteGlu_2–7_ and MeFox-PteGlu_7_ were injected to determine the fragmentation patterns of those.

## 5. Conclusions

With the experiments performed, we could demonstrate why vitamins and especially folate vitamers have not been discussed in metabolomics studies yet. We found several reasons for this observation:

- Relatively low concentration of analytes (µM range);
- Insufficient folate extraction in typical un-targeted set-ups (ice-cold extraction);
- Insufficient dissolution of folates in the organic solvent mixtures usually used for sample preparation;
- Insufficient optimization of (SPE) purification procedures;
- Loss of intensity for the more general SPE procedures applied (RP phase and polymeric resin);
- Low ionizing potential using 0.1% formic acid concentration in the mobile phase of UHPLC separation coupled to MS analysis;
- Interconversion and oxidation reactions need to be taken into account.

The tested shotgun approaches confirmed the difficulties of extracting folate vitamers with classical un-targeted approaches. First, folate vitamers occur in low abundance of µM even in an organism known to be characterized by high folate contents such as baker’s yeast. Second, conditions typically applied in un-targeted studies might not be effective enough for sufficient folate extraction. Ice-cold temperatures and usage of organic solvents were shown not to be the ideal conditions for folate extraction. Furthermore, matrix effects might lead to further ion suppression of folates already ionizing with low intensities. To increase the ionizing potential of folates, formic acid concentrations in mobile phases used for UHPLC separation should be raised to 1%. To reduce matrix effects, SPE procedures should be applied and optimized for the ideal extraction of folate vitamers. Here, the use of anion exchange material enabled additional identification based on MS^2^ spectra due to the higher intensities of precursor ions.

Last, the occurrence of interconversion and oxidation reactions of folate vitamers should be considered when applying different SPE protocols. We observed decreased contents of formyl derivatives depending on the purification protocol which we could ascribe to interconversion reactions not covered by the applied SIDA method.

The main polyglutamates identified were 5-CH_3_-H_4_PteGlu_6–8_, 5-CHO-H_4_PteGlu_6–8_, H_4_PteGlu_6–8_, 5,10-CH^+^-H_4_PteGlu_6–8_, 10-CHO-PteGlu_6–8_, PteGlu_6–8_ and the oxidation product MeFox-Glu_6–8_. Quantitative results revealed similar folate contents in yeast around 4188 to 4398 µg/100 g as previously reported [70,71,72].

## Figures and Tables

**Table 1:** Substitution of main folate vitamers known so far.

Trivial Name	Abbreviation	R_1_	R_2_	R_3_
Pteroylglutamic acid	PteGlu	–N^5^=	H	–N^8^=
7,8-dihydrofolate	7,8-H_2_folate	H	H	–N^8^=
5,6,7,8-tetrahydrofolate	5,6,7,8-H_4_folate	H	H	H
5-methyl-tetrahydrofolate	5-CH_3_-H_4_folate	CH_3_	H	H
5-methyl-dihydrofolate	5-CH_3_-H_2_folate	CH_3_	H	–N^8^=
5-formyl-tetrahydrofolate	5-CHO-H_4_folate	CHO	H	H
10-formyl-tetrahydrofolate	10-CHO-H_4_folate	H	CHO	H
10-formyl-dihydrofolate	10-CHO-H_2_folate	H	CHO	–N^8^=
10-formyl-pteroic acid	10-CHO-PteGlu	–N^5^=	CHO	–N^8^=
5,10-methenyl-tetrahydrofolate	5,10-CH^+^-H_4_folate	–N^5^–CH^+^=N^10^–		H
5,10-methylene-tetrahydrofolate	5,10-CH_2_-H_4_folate	–N^5^–CH_2_–N^10^–		H

**Table 2:** Overview of different purification procedures by solid phase extraction applied during our studies.

Procedure	SAXa	SAXb	HLB	C18
pH of sample	5	5	2	2
Elution solution	0.1 M NaAc + 5% NaCl, 1% ascorbic acid, 0.1% DTT (dithiothreitol)	20% ACN + 5% formic acid	MeOH	20% ACN + 0.1% formic acid

## Supplementary Materials

The supplementary materials are available online at https://www.mdpi.com/2218-1989/10/8/301/s1.
