# Supplementary material for: Comprehensive Vitamer Profiling of Folate Mono- and Polyglutamates in Baker’s Yeast (Saccharomyces cerevisiae) as a Function of Different Sample Preparation Procedures
Source: Metabolites. 2020 Jul 23;10(8):301. doi: 10.3390/metabo10080301 (PMC7464241; doi:10.3390/metabo10080301)
Supplement: Supplementary file 1 [file metabolites-10-00301-s001.zip › metabolites-851343-sup-final-v3/metabolites-851343-supplementary-for conversion.pdf]

metabolites

MDPI

Metabolites

Supporting Information

Comprehensive Vitamer Profiling of Folate Mono- and Polyglutamates in Baker's Yeast (*Saccharomyces cerevisiae*) as a Function of Different Sample Preparation Procedures

Lena Gmelch [1], Daniela Wirtz [1], Michael Witting [1,2], Nadine Weber [1], Lisa Striegel [1], Philippe Schmitt-Kopplin [1,2,*] and Michael Rychlik [1,2,*]

[1] Chair of Analytical Food Chemistry, Technical University of Munich, 85354 Freising-Weihenstephan, Germany; lena.gmelch@tum.de (L.G.); ga65way@mytum.de (D.W.); michael.witting@helmholtz-muenchen.de (M.W.); nadine1.weber@tum.de (N.W.); lisa.striegel@tum.de (L.S.)

[2] Research Unit BioGeoChemistry, Helmholtz Zentrum Munich, 85764 Neuherberg, Germany;

* Correspondence: schmitt-kopplin@helmholtz-muenchen.de (P.S.-K.); michael.rychlik@tum.de (M.R.)

1. Application of Different Shotgun Approaches

Extraction with different solvent compositions and varying extraction conditions was tested. Tested solvent compositions were 80% methanol, 50% acetonitrile, methanol/acetonitrile/water (40/40/20, *v*/*v*/*v*), and methanol/isopropanol/water (1/1/1, *v*/*v*/*v*). For each of the tested conditions, 1 mL of extraction solvent was added to 50 mg lyophilized yeast sample and a cell disruption procedure applied by using a Precellys tissue homogenizer (Precellys Evolution, Bertin GmbH, Frankfurt, Germany). Samples were sonicated for 10 min and centrifuged (10 min at 20,000× *g* at 4 °C). The residue was treated in the same way for further extraction. Supernatants were combined, evaporated to dryness and resuspended in 500 µL of the respective extraction solvent used beforehand. Samples were either cooled on ice, kept at room temperature or heated up to 80 °C during sonication for each of the tested extraction solvents. Thus, we investigated twelve different extraction protocols in total. No MS^1 features assignable to an already known folate vitamer was found in any of the analyzed samples. Insufficient folate extraction using solvent mixtures as well as matrix effects were supposed to be responsible for this lack of folate detection. Adjusting the sample preparation for better folate extraction by boiling in buffer solution as well as sample purification applying solid phase extraction (SPE), detection of folate vitamers was possible. Using strong anion exchange cartridges (SAX) in combination with a buffer solution used for sample extraction, a clear reduction of matrix effects could be observed as depicted by the (red) base peak chromatogram (BPC) in Figure S1 compared to the (black) BPC of the shotgun approach.

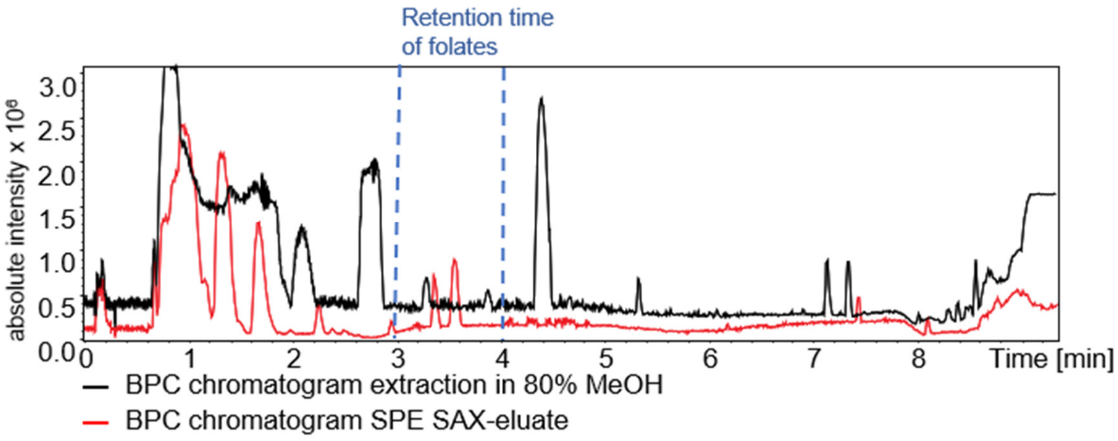

Figure S1. Base Peak Chromatogram (BPC) of baker's yeast extracted with 80% methanol (black line) and after purification by solid phase extraction (SPE) using strong anion exchange (SAX) cartridges and sample elution with a buffer solution highlighting the reduction of matrix effects. Samples were measured by UHPLC-Q-ToF-MS.

2. Selection of Solid Phase Extraction (SPE) Materials for Sample Purification

In a preliminary study design, different SPE materials were tested for their applicability in folate analysis. Cartridge materials were chosen based on their retention mechanism being suitable for folate analytes. The efficiency of folate extraction was compared to the standard procedure using strong anion exchange (SAX) material and elution with a buffer solution. Tested SPE materials were SAX, phenyl, amino propyl, C18, HLB, and anion mixed mode cartridges. In a first approach, different folate monoglutamate standard solutions (5-CH_3-H_4folate, 5-CHO-H_4folate, H_4folate, and 10-CHO-PteGlu) were used to analyze the general retention behavior of folate analytes in dependence on the cartridge chemistry used for purification. A pre-selection of cartridges was made by a simple comparison of peak areas of the folate analytes in the extracts analyzed by means of LC-MS/MS after separation on C18-material Peak areas of folate analytes purified by SAX-cartridges using a buffer solution for elution were set to an efficiency of 100%. Those cartridges providing best relative recoveries were selected for further optimization. Table S1 summarizes the SPE materials tested and highlights those cartridges which were selected for further optimization. It was shown that reversed phase materials needed the acidification of eluates for complete protonation and thus effective retention of folates (better efficiency of C18 cartridges when adjusting the pH to pH 3.4 compared to loading at pH 5). Anion exchange materials (NH_2 and SAX) enabled sample loading without adjustment of pH. However, only SAX cartridges provided convincing folate contents in the final eluates yet needed acidic conditions for the elution step.

Table S1. Tested solid phase extraction materials for the purification of the folate monoglutamate standards 5-CH_3-H_4folate, 5-CHO-H_4folate, H_4folate, and 10-PteGlu. The efficiency of the elution was calculated by comparison of peak areas after analysis by LC-MS/MS for each analyte. Peak areas after purification by SAX-cartridges using a buffer solution for elution were set to 100%. The efficiency range covers the margin in which the individual standards fell. Selected cartridge materials for further optimization are highlighted by "x".

SPE Material	pH of Sample	Elution Solution	Efficiency of Folate Monoglutamate Elution	Selection for Further Optimization
Amino propyl (NH_2)	5	Methanol + 1% NH_3	0.1–2.0%	
Amino propyl (NH_2)	5	Methanol + 1% formic acid	0.0–0.4%	
C18	3.4	50% methanol + 0.1% formic acid	2.2–48%	x
C18	5	50% methanol + 0.1% formic acid	0.3–2.7%	
Hydrophilic lipophilic balanced (HLB)	2	methanol	8.4–85%	x
Mixed anion exchange (MAX)	5	Methanol + 1% formic acid	0.0–0.2%	
phenyl	3.4	40% acetonitrile	0.0–2.0%	
Strong anion exchange (SAX)	5	50% methanol + 1% formic acid	20–53%	x

In a further step, baker's yeast samples were analyzed to account for the efficiency of polyglutamate purification. For this purpose, samples were spiked with the internal standard (ISTD) $^{13}C_5$-5-CH_3-H_4folate prior to folate extraction to account for loss during sample preparation. Samples were measured by means of LC-MS/MS. However, separation of polyglutamates was conducted on a different C18-material (YMC-Pack Pro C18 RP 150 x 3.0 mm, 3 µm, YMC, Dinslaken, Germany). Water and acetonitrile containing 1% of formic acid each served as mobile phases A and B, respectively. The column was kept at 30 °C, the flow rate was set to 0.3 mL/min, the injection volume was 20 µL. The gradient for separation was as follows: 0 min, 5% B; 0–2 min, 10 % B; 2–11 min, 10% B; 11–15 min, 15% B; 15–17 min, 90% B; 17–19 min; 90% B; 19–21 min, 5% B; 21–26 min, 5% B. Instrument settings are listed in Supplementary Table S2. Peak ratios of the extracted folates were calculated in comparison to the spiked standard. A stepwise optimization of the selected SPE materials was performed to elute as many vitamers as possible in as many polyglutamate states as possible. Figure S2 shows the elution efficiency of the main folate polyglutamates (heptaglutamates) for the optimized SPE procedures.

Table S2. MRM transitions of several 5-CH_3-H_4PteGlu polyglutamates and the heptaglutamates of 5-CHO-H_4folate, H_4folate, 5,10-CH^+-H_4folate, 10-CHO-PteGlu and PteGlu analyzed with LC-MS/MS.

Compound	Precursor [*m*/*z*]	Product [*m*/*z*]	Dwell Time [ms]	Q1 Pre Bias [V]	CE [V]	Q3 Pre Bias [V]	Retention Time Window [min]
5-CH_3-H_4PteGlu$_1$	460.20	313.20	70.0	−13.0	−20.0	−17.0	7.8–8.2
	460.20	180.15	70.0	−13.0	−37.0	−14.0	
	460.20	194.25	70.0	−23.0	−33.0	−22.0	
5-CH_3-H_4PteGlu$_2$	589.30	313.20	70.0	−28.0	−25.0	−25.0	8.0–8.2
	589.30	180.15	70.0	−36.0	−65.0	−25.0	
5-CH_3-H_4PteGlu$_3$	359.65	313.20	70.0	−20.0	−15.0	−22.0	8.2–8.6
	359.65	180.15	70.0	−20.0	−33.0	−20.0	
5-CH_3-H_4PteGlu$_4$	424.15	313.20	70.0	−15.0	−20.0	−21.0	8.8–9.2
	424.15	180.15	70.0	−15.0	−40.0	−13.0	

5-CH_3-H_4PteGlu$_5$	488.65	313.20	70.0	−30.0	−25.0	−34.0	9.2–9.6
	488.65	180.15	70.0	−30.0	−62.0	−20.0	
5-CH_3-H_4PteGlu$_6$	553.20	313.20	70.0	−18.0	−20.0	−34.0	9.5–10.0
	553.20	180.15	70.0	−18.0	−45.0	−20.0	
5-CH_3-H_4PteGlu$_7$	617.70	313.20	70.0	−34.0	−20.0	−36.0	9.8–10.2
	617.70	180.15	70.0	−34.0	−53.0	−20.0	
5-CH_3-H_4PteGlu$_8$	682.20	313.20	70.0	−12.0	−16.0	−21.0	10.0–10.4
	682.20	180.15	70.0	−12.0	−36.0	−13.0	
5-CH_3-H_4PteGlu$_9$	746.80	313.20	70.0	−34.0	−20.0	−36.0	10.3–10.6
	746.80	180.15	70.0	−34.0	−53.0	−20.0	
5-CH_3-H_4PteGlu$_{10}$	541.20	313.20	70.0	−34.0	−20.0	−36.0	10.5–11.2
	541.20	180.15	70.0	−34.0	−53.0	−20.0	
[$^{13}C_5$]-5-CH_3-H_4folate	465.30	313.20	70.0	−13.0	−20.0	−17.0	7.8–8.2
	465.30	180.15	70.0	−13.0	−37.0	−14.0	
	465.30	194.25	70.0	−23.0	−33.0	−22.0	
5-CHO-H_4PteGlu$_7$	624.60	327.10	70.0	−34	−20	−36	13.2–14.2
	624.60	299.10	70.0	−34	−53	−20	
H_4PteGlu$_7$	610.65	299.20	70.0	−34	−20	−36	8.8–9.2
	610.65	299.20	70.0	−34	−53	−20	
5,10-CH^+-H_4PteGlu$_7$	615.70	412.00	70.0	−34	−20	−36	10.4–11.0
	615.70	282.00	70.0	−34	−53	−20	
10-CHO-PteGlu$_7$	622.60	295.00	70.0	−34	−20	−36	13.2–14.2
	622.60	176.10	70.0	−34	−53	−20	
PteGlu$_7$	608.60	295.10	70.0	−34	−20	−36	13.6–14.3
	608.60	176.30	70.0	−34	−53	−20	

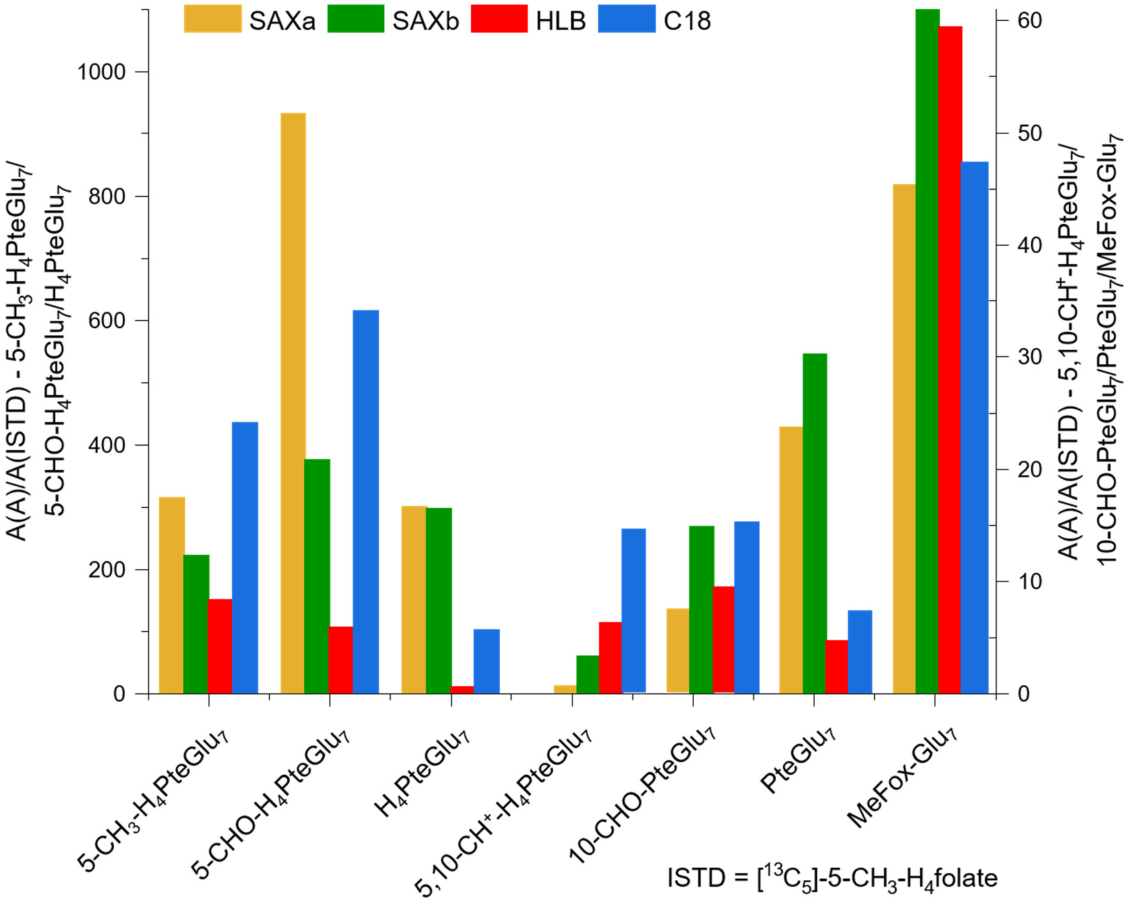

Figure S2. Semi-quantitative analysis of the folate vitamers detectable in baker's yeast samples after optimization of the solid phase extraction procedures with SAXa: Strong Anion Exchange (SAX) and elution with elution buffer; SAXb: SAX and elution with 20% acetonitrile and 5% formic acid; HLB: Hydrophilic Lipophilic Balanced; C18. Samples were measured with LC-MS/MS and relative peak areas of the heptaglutamate vitamers calculated after normalization for the peak area of the internal standard (ISTD) [$^{13}C_5$]-5-CH_3-H_4folate. Relative ratios for 5-CH_3-H_4PteGlu$_7$, 5-CHO-H_4PteGlu, and H_4PteGlu$_7$ are shown on the left axis whereas results for 5,10-CH^+-H_4PteGlu$_7$, 10-CHO-PteGlu$_7$, PteGlu$_7$, and MeFox-Glu$_7$ are shown on the right axis.

3. Optimization of the UHPLC-Method for Folate Analysis by UHPLC-Q-ToF-MS

3.1. Selection of the UHPLC-Column

Different UHPLC columns were tested for their applicability for folate analysis:

- Acquity UPLC BEH C18, 1.7 µm, 2.1 × 100 mm, Waters, Eschborn, Germany
- Acquity UPLC CSH C18, 1.7 µm, 2.1 × 1000 mm, Waters, Eschborn, Germany
- Cortecs UPLC C18, 1.6 µm, 2.1 × 100 mm, Waters, Eschborn, Germany
- Cortecs UPLC C18+, 1.6 µm, 2.1 × 100 mm, Waters, Eschborn, Germany
- Restek Raptor™, ARC-18, 1.8 µm, 2.1 × 100 mm, Restek GmbH, Bad Homburg, Germany

Of all the tested reversed phase-columns, Restek Raptor™, ARC-18 material provided best peak shapes for each of the tested monoglutamate standards (5-CH_3-H_4folate, 5-CHO-H_4folate, H_4folate, 10-CHO-PteGlu, and PteGlu) as well as for the synthesized polyglutamates 5-CH_3-H_4PteGlu$_{2-7}$ for the tested gradients and, therefore, was chosen for further experiments.

3.2. Selection of the Gradient Program

Water and acetonitrile containing 0.1% formic acid each served as solvent A and B, respectively. The flow rate was set to 0.3 mL min^{-1}. Two different gradient programs were tested. Gradient 1 represented a standard operation procedure, while gradient 2 was adapted from the gradient used for quantification using LC-MS/MS. Tested gradients were as follows:

- Gradient 1: pre-run time 3.7 min; 0.0 min 5% B, 0–1.12 in 5% B, 1.13–6.41 min 0.4% B, 6.42–10.01 min 0.4% B.
- Gradient 2: pre-run time 3.7 min; 0.0 min 3% B; 0–1 min, 3% B; 1–2.25 min, 10% B; 2.25–3.5 min, 10% B; 3.5–6.5 min, 50% B; 6.5–7 min 50% B; 7–7.3 min 99.9% B; 7.3–9 min, 99.9% B; 9–9.3 min, 3% B.

Gradient 2 provided better separation of the tested folate standards and therefore was chosen for all the subsequent measurements.

3.3. Selection of the Mobile Phase

Due to poor ionization of the synthesized polyglutamate standards (especially those above a molecular weight of 1000 Da), the concentration of formic acid in the solvents was raised to 1%. An increased concentration of acid ensured ideal ionization of poyglutamates with a molecular weight > 1000 Da and therefore was used for all subsequent measurements.

3.4. Investigation of the Fragmentation Behavior of Synthesized Polyglutamate Standards

To identify the retention behavior after chromatographic separation and fragmentation behavior of folate polyglutamates, MeFox-Glu$_7$ was synthesized according to the procedure of Ringling et al. [1] and measured by UHPLC-Q-ToF-MS. The monoglutamate standard MeFox was analyzed in the same way and results were compared. Results of the comparison between MeFox and MeFox-Glu$_7$ are shown in Supplementary Figure S3.

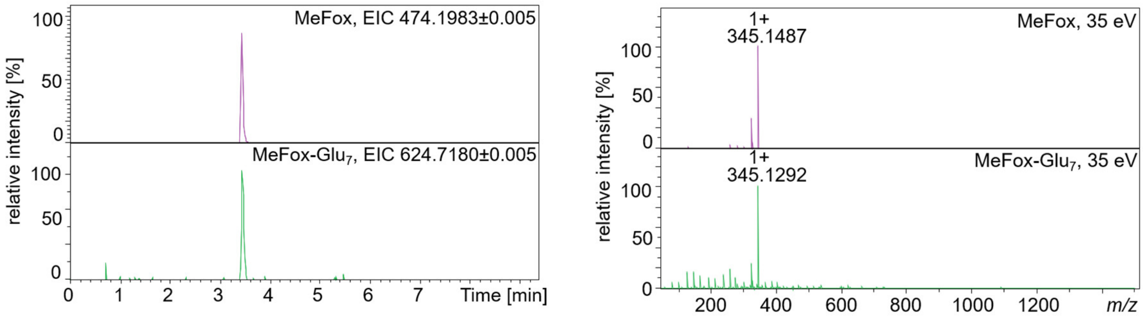

(**a**) (**b**)

Figure S3. MeFox and MeFox-Glu$_7$ measured on the UHPLC-Q-ToF-MS after chromatographic separation on a Restek Raptor™ ARC-18 column (1.8 µm, 100x2.1 mm): (**a**) Extracted Ion Chromatograms (EIC) of the folate standards; (**b**) MS^2 spectra of MeFox and MeFox-Glu$_7$ at CE = 35 eV.

4. Percentage Distribution of Folate Polyglutamates after Analysis by UHPLC-Q-ToF-MS

The percentage distribution of folate polyglutamates after analysis by UHPLC-Q-ToF-MS was calculated for each group of vitamer as the percentage of the total sum of intensities for the four applied purification procedures. Supplementary Table S3 gives detailed numbers for the 5-CH_3-H_4folate polyglutamates whereas Supplementary Figure S4 gives a graphical overview for each group of vitamer.

Table S3. Distribution of 5-CH_3-H_4folate polyglutamates as percentage of the total sum of intensities for different SPE purification procedures applied with SAXa: Strong Anion Exchange (SAX) and elution with elution buffer; SAXb: SAX and elution with 20% acetonitrile and 5% formic acid; HLB: Hydrophilic Lipophilic Balanced; C18. Samples were measured by UHPLC-Q-ToF-MS; n.d. = not detectable.

	5-CH_3-H_4PteGlu$_n$ [% of Total Sum of Intensities]								
	n = 1	n=2	n = 3	n = 4	n = 5	n = 6	n = 7	n = 8	n = 9
SAXa	n.d.	0.14 ± 0.02	0.15 ± 0.02	0.37 ± 0.05	0.23 ± 0.02	13.0 ± 0.9	65.9 ± 5.4	19.4 ± 1.7	0.88 ± 0.09
SAXb	0.03 ± 0.01	0.29 ± 0.06	0.22 ± 0.02	0.52 ± 0.08	0.29 ± 0.04	14.2 ± 1.4	61.2 ± 6.1	22.3 ± 2.5	0.91 ± 0.32
HLB	0.19 ± 0.03	0.51 ± 0.05	0.91 ± 0.13	0.38 ± 0.10	0.18 ± 0.04	8.64 ± 1.10	65.4 ± 9.0	22.6 ± 3.7	1.20 ± 0.14
C18	0.80 ± 0.18	0.50 ± 0.19	1.78 ± 0.44	1.56 ± 0.40	1.84 ± 0.10	8.36 ± 1.02	64.0 ± 7.8	20.1 ± 1.8	1.09 ± 0.07

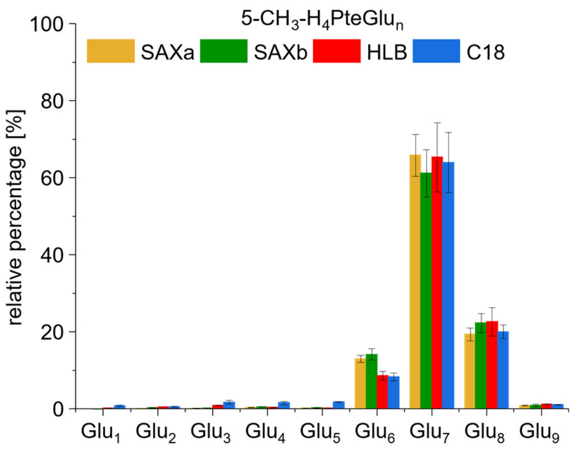

(**a**)

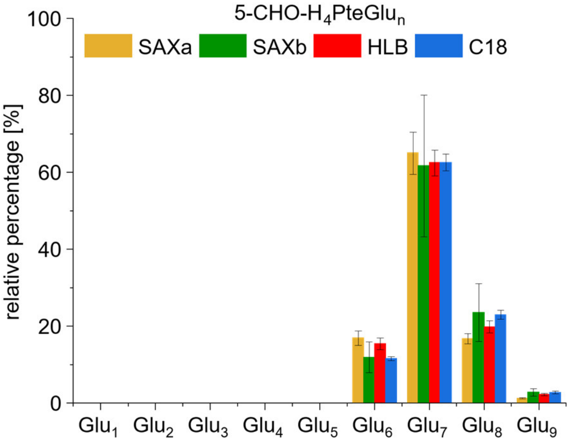

(**b**)

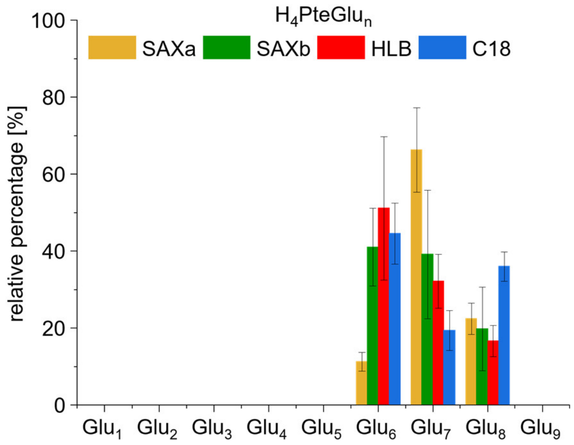

(**c**)

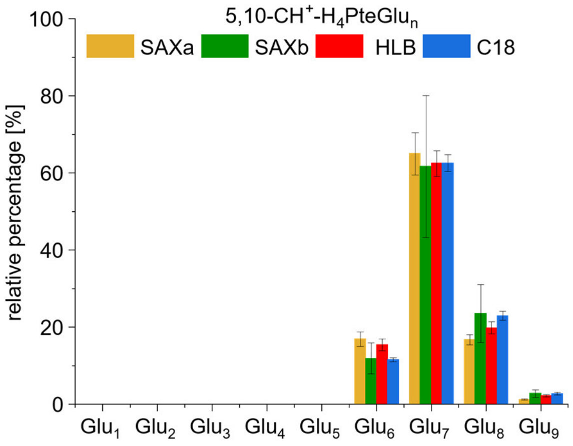

(**d**)

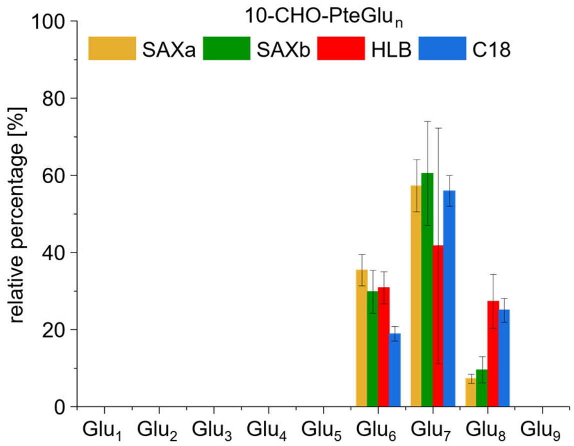

(**e**)

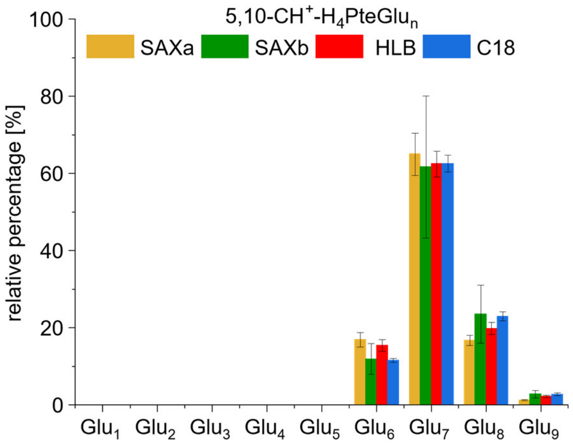

(**f**)

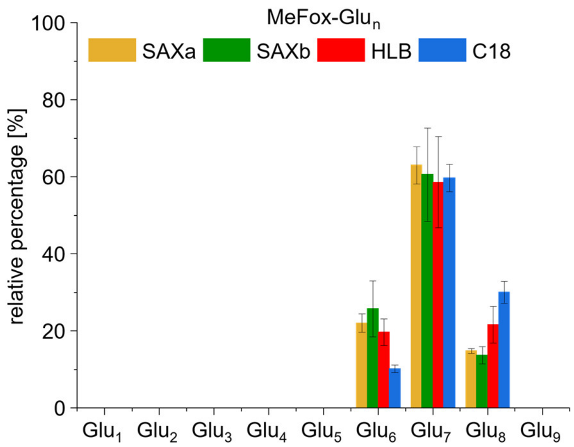

(**g**)

Figure S4. Qualitative analysis of the folate vitamers in baker's yeast samples purified by different solid phase extraction materials and measured with UHPLC-Q-ToF-MS after purification with SAXa: Strong Anion Exchange (SAX) and elution with elution buffer; SAXb: SAX and elution with 20% acetonitrile and 5% formic acid; HLB: Hydrophilic Lipophilic Balanced; C18. Relative polyglutamate

distribution is shown for each of the analyzed vitamers. The sum of intensities of all determined polyglutamates for each vitamer was equaled to 100%: (**a**) relative 5-CH_3-H_4PteGlu$_n$ distribution (**b**) relative 5-CHO-H_4PteGlu$_n$ distribution (**c**) relative H_4PteGlu$_n$ distribution (**d**) relative 5,10-CH^+-H_4PteGlu$_n$ distribution (**e**) relative 10-CHO-PteGlu$_n$ distribution (**f**) PteGlu$_n$ distribution (**g**) relative MeFox-Glu$_n$ distribution.

5. Adaptation of the DDA (Data Dependent Acquisition) Mode Method for Fragmentation of Folate Polyglutamates by Q-ToF-MS

The DDA method applied for analysis of folate polyglutamates needed to be adapted to enable generation of fragmentation spectra for the folate polyglutamates. The adapted preference list can be found in Supplementary Table S4.

Table S4. Preference list of DDA method applied for UHPLC-Q-ToF-MS measurements. The minimum intensity for precursor selection was set to 1000.

m/*z* Range	*m*/*z* Range Continued	*m*/*z* Range Continued
292.6–293.6	486.17–487.17	624.21–625.21
294.62–295.62	488.18–489.18	672.72–673.72
301.61–302.61	495.17–496.17	674.74–675.74
357.12–358.12	543.68–544.68	679.73–680.73
359.14–360.14	545.69–546.69	681.74–682.74
366.13–367.13	550.69–551.69	686.72–687.72
416.65–417.65	552.70–553.70	688.73–689.73
421.64–422.64	557.68–558.68	737.24–738.24
423.66–424.66	559.69–560.69	739.26–740.26
430.65–431.65	608.2–609.2	744.25–745.25
455.66–456.66	611.22–612.22	746.27–747.27
459.69–460.69	615.21–616.21	751.24–752.24
473.67–474.67	617.22–618.22	753.26–754.26
481.17–482.17	622.20–623.20	

6. Summary of Detected and Further Identified Folate Vitamers in Baker's Yeast

Folate polyglutamate detection for the different purification procedures was based on the MS-features obtained after analysis by UHPLC-Q-ToF-MS. Further identification was accepted based on corresponding fragmentation spectra. Supplementary Table S5 gives an overview of the obtained results.

Table S5. Summary of the detected and further identified folate vitamers in baker's yeast analyzed with UHPLC-Q-ToF-MS. Identification was achieved by signal detection in the MS^1 spectra at the corresponding retention time known from measurements of monoglutamate standards. Further identification was achieved by fragmentation information by MS^2 spectra.

		**Formula**	**RT [min]**	***m/z***	**Identified with MS^1**				**Identified with MS^2**			
					**SAXa**	**SAXb**	**HLB**	**C18**	**SAXa**	**SAXb**	**HLB**	**C18**
5-CH_3-H_4PteGlu$_n$	*n* = 1	$C_{20}H_{25}N_7O_6$	3.2	460.1939	x	x	x					
	n = 2	$C_{25}H_{32}N_8O_9$	3.2	295.1219	x	x	x	x	x		x	
	n = 3	$C_{30}H_{39}N_9O_{12}$	3.2	359.6429	x	x	x	x	x			
	n = 4	$C_{35}H_{46}N_{10}O_{15}$	3.3	424.1643	x	x	x	x	x	x		
	n = 5	$C_{40}H_{53}N_{11}O_{18}$	3.3	488.6855	x	x	x	x	x	x		
	n = 6	$C_{45}H_{62}N_{12}O_{19}$	3.3	553.2064	x	x	x	x	x	x	x	x
	n = 7	$C_{50}H_{67}N_{13}O_{24}$	3.4	617.7276	x	x	x	x	x	x	x	x
	n = 8	$C_{55}H_{74}N_{14}O_{27}$	3.4	682.2501	x	x	x	x	x	x	x	x
	n = 9	$C_{60}H_{81}N_{15}O_{30}$	3.4	746.7706	x	x	x	x	x	x	x	
5-CHO-H_4PteGlu$_n$	*n* = 1	$C_{20}H_{23}N_7O_7$										
	n = 2	$C_{25}H_{30}N_8O_{10}$										
	n = 3	$C_{30}H_{37}N_9O_{13}$										
	n = 4	$C_{35}H_{44}N_{10}O_{16}$										
	n = 5	$C_{40}H_{51}N_{11}O_{19}$										
	n = 6	$C_{45}H_{58}N_{12}O_{22}$	3.8	560.1969	x	x	x	x	x	x		
	n = 7	$C_{50}H_{65}N_{13}O_{25}$	3.8	624.7185	x	x	x	x	x	x	x	x
	n = 8	$C_{55}H_{72}N_{14}O_{28}$	3.9	689.2379	x	x	x	x	x	x	x	x
	n = 9	$C_{60}H_{79}N_{15}O_{31}$										
H_4PteGlu$_n$	*n* = 1	$C_{19}H_{23}N_7O_6$										
	n = 2	$C_{24}H_{30}N_8O_9$										
	n = 3	$C_{29}H_{37}N_9O_{12}$										
	n = 4	$C_{34}H_{44}N_{10}O_{15}$										
	n = 5	$C_{39}H_{51}N_{11}O_{18}$										
	n = 6	$C_{44}H_{58}N_{12}O_{21}$	3.2	546.198	x	x	x	x				
	n = 7	$C_{49}H_{65}N_{13}O_{24}$	3.2	610.2698	x	x	x	x	x	x		
	n = 8	$C_{54}H_{72}N_{14}O_{27}$	3.3	675.2405	x	x	x	x	x			
	n = 9	$C_{59}H_{79}N_{15}O_{30}$										

PteGlu$_n$	n = 1	$C_{19}H_{19}N_7O_6$										
	n = 2	$C_{24}H_{26}N_8O_9$										
	n = 3	$C_{29}H_{33}N_9O_{12}$										
	n = 4	$C_{34}H_{40}N_{10}O_{15}$										
	n = 5	$C_{39}H_{47}N_{11}O_{18}$										
	n = 6	$C_{44}H_{54}N_{12}O_{21}$	3.9	544.1839	x	x	x	x				
	n = 7	$C_{49}H_{61}N_{13}O_{24}$	3.9	608.7039	x	x	x	x	x	x		
	n = 8	$C_{54}H_{68}N_{14}O_{27}$	3.9	673.2237		x	x	x				
	n = 9	$C_{59}H_{75}N_{15}O_{30}$										
10-CHO-PteGlu$_n$	n = 1	$C_{20}H_{19}N_7O_7$										
	n = 2	$C_{25}H_{26}N_8O_{10}$										
	n = 3	$C_{30}H_{33}N_9O_{13}$										
	n = 4	$C_{35}H_{40}N_{10}O_{16}$										
	n = 5	$C_{40}H_{47}N_{11}O_{19}$										
	n = 6	$C_{45}H_{54}N_{12}O_{22}$	3.7	558.1809	x	x	x	x	x	x		
	n = 7	$C_{50}H_{61}N_{13}O_{25}$	3.8	622.7019	x	x	x	x	x	x	x	x
	n = 8	$C_{55}H_{68}N_{14}O_{28}$	3.8	687.2218	x	x	x	x	x	x		
	n = 9	$C_{60}H_{77}N_{15}O_{31}$										
5,10-CH$^+$-H$_4$PteGlu$_n$	n = 1	$C_{21}H_{22}N_7O_6$										
	n = 2	$C_{26}H_{29}N_8O_9$										
	n = 3	$C_{31}H_{36}N_9O_{12}$										
	n = 4	$C_{36}H_{43}N_{10}O_{15}$										
	n = 5	$C_{41}H_{50}N_{11}O_{18}$										
	n = 6	$C_{46}H_{57}N_{12}O_{21}$	3.4	551.1914	x	x	x	x	x	x		
	n = 7	$C_{51}H_{64}N_{13}O_{24}$	3.5	615.7128	x	x	x	x	x	x	x	x
	n = 8	$C_{56}H_{71}N_{14}O_{27}$	3.5	680.2333	x	x	x	x	x	x		
	n = 9	$C_{61}H_{78}N_{15}O_{30}$	3.5	744.7537	x	x	x	x	x			
MeFox-Glu$_n$	n = 1	$C_{20}H_{23}N_7O_7$										
	n = 2	$C_{25}H_{30}N_8O_{10}$										
	n = 3	$C_{30}H_{37}N_9O_{13}$										
	n = 4	$C_{35}H_{44}N_{10}O_{16}$										
	n = 5	$C_{40}H_{51}N_{11}O_{19}$										
	n = 6	$C_{45}H_{58}N_{12}O_{22}$	3.5	560.1967	x	x	x	x	x	x		
	n = 7	$C_{50}H_{65}N_{13}O_{25}$	3.5	624.7159	x	x	x	x	x	x		
	n = 8	$C_{55}H_{74}N_{14}O_{26}$	3.5	689.2375	x	x	x	x	x	x		
	n = 9	$C_{60}H_{79}N_{15}O_{31}$										

7. Validation of the Quantification of MeFox after Enzymatic Deconjugation

In order to investigate the influence of different purification procedures on the folate pattern, we aimed at quantifying different known oxidation products (shown in Figure 3a and 3b) after enzymatic deconjugation into the respective monoglutamate forms. Therefore, we included *s*-Pyrazino-triazine (commonly known as MeFox) into the already established method for the quantitation of the total folate content [2]. The MRM scan parameters obtained after method optimization can be found in Supplementary Table S6a. Method validation was performed according to the procedure of Hädrich and Vogelgesang [3].

7.1. Determination of the Response Curves for the Analysis of MeFox after Enzymatic Deconjugation

For the response curve of MeFox, PteGlu was used as internal standard (ISTD) on the HPLC-DAD. For the response curve at the LC-MS/MS system, [$^{13}C_5$]-5-CHO-H$_4$folate served as ISTD. A constant amount of internal standard was mixed with varying amounts of analyte (A) to give a molar ration of [n(A)/n(ISTD)] ranging between 0.48 and 7.62 for the HPLC-DAD and 0.05 and 8.49 for the

LC-MS/MS system. Linear regression was confirmed by the linearity test of Mandel [4] for both calibration functions. The calibration curve was linear for a molar ratio n(A)/n(ISTD) between 0.32 and 5.27 at the HPLC-DAD ($y = 1.4468x - 0.0001$; $R^2 = 0.9999$), and between 0.05 and 8.49 at the LC-MS/MS ($y = 0.4900x + 0.0008$, $R^2 = 0.9999$), respectively.

7.2. Determination of the Limit of Detection (LOD), Limit of Quantification (LOQ), Precision and Recovery for MeFox after Enzymatic Deconjugation

The limit of detection and limit of quantification of MeFox were determined according to the method of Hädrich and Vogelgesang [3]. Therefore, a MeFox free matrix had to be found. Since all methods for the destruction of folates will lead to the formation of MeFox, a yeast-like matrix was mixed consisting of cellulose (94%), L-leucine (1.30%), L-lysine (1.26%), L-valine (1.01%), L-isoleucine (0.89%), L-threonine (0.82%) and oleic acid (0.7%) in a composition naturally occurring in yeast. After lyophilization, the blank matrix was spiked with four different levels of MeFox with the lowest level being slightly above the expected LOD (three times the background noise) and the highest level being 10-fold higher. The spiked matrices were analyzed in triplicate according to the procedure mentioned above for the total folate analysis. The concentration was determined for each spike level and the results correlated with the spike amounts. The correlation was used to determine LOD and LOQ according to the method of Hädrich and Vogelgesang [3]. Inter-day precision for the analysis of MeFox was determined by three independent analyses of a yeast sample in quadruples over a period of three weeks. Inter-injection precision was determined by injecting the same sample fifteen times in a row. Triplicate analysis of a yeast sample gave the intra-day precision.

The determined results are listed in Table S2. The LOD was 0.80 µg/100 g, the LOQ was 2.53 µg/100 g. These values were slightly higher than those for the other analytes. The inter-injection precision for an analyzed yeast sample was 4.6%, the intra-day precision 2.2%, and the inter-day precision 6.1%, respectively. The recovery results were obtained after spiking of a matrix consisting of cellulose and different amino acids with three different levels of MeFox. Recoveries ranged from 97.6% to 113%, and thus were in the same range as recoveries for the other analytes previously reported [2].

Table S6. Validation data for the stable isotope dilution assay of MeFox after enzymatic deconjugation. The LOD, LOQ, and recovery values were determined in a matrix of cellulose and different amino acids. The precision values were determined with baker's yeast samples.

LOD [µg/100 g]	LOQ [µg/100 g]	Precision (n = 3) [RSD%]			Recovery [%]		
		Inter-Injection	Intra-Day	Inter-Day	Spiking Level 1	Spiking Level 2	Spiking Level 3
**0.80**	2.53	4.6	2.2	6.1	97.6	111	113

7.3. Inclusion of the MRM (Multiple Reaction Monitoring) transitions of MeFox in the Method for the Quantification of Total Folate contents by UHPLC-MS/MS

MRM transitions obtained for the analyte MeFox were included into the method for quantification of the total folate content by UHPLC-MS/MS. MRM transitions can be found in Supplementary Table S7.

Table S7. MRM transitions of several monoglutamates analyzed with UHPLC-MS/MS.

Compound	Precursor [*m*/*z*]	Product [m/z]	Dwell Time [ms]	Q1 Pre Bias [V]	CE [V]	Q3 Pre Bias [V]	Retention Time Window [min]
PteGlu	442.30	295.15	70.0	−13.0	-16.0	-16.0	5.6–5.8
	442.30	176.20	70.0	−13.0	−37.0	−20.0	
	442.30	120.05	70.0	−13.0	−35.0	−14.0	

[13C5]-PteGlu	447.20	295.15	70.0	−13.0	−16.0	−16.0	5.6–5.8
	447.20	176.20	70.0	−13.0	−37.0	−20.0	
	447.20	120.05	70.0	−13.0	−35.0	−14.0	
H4folate	446.00	299.20	70.0	−22.0	−20.0	−16.0	3.3–3.5
	446.00	120.10	70.0	−22.0	−37.0	−14.0	
	446.00	166.15	70.0	−22.0	−41.0	−19.0	
[13C5]-H4folate	451.30	299.20	70.0	−22.0	−20.0	−16.0	3.3–3.5
	451.30	120.10	70.0	−22.0	−37.0	−14.0	
	451.30	166.15	70.0	−22.0	−41.0	−19.0	
5-CH3–H4folate	460.20	313.20	70.0	−13.0	−20.0	−17.0	3.6–3.8
	460.20	180.15	70.0	−13.0	−37.0	−14.0	
	460.20	194.25	70.0	−23.0	−33.0	−22.0	
[13C5]-5-CH3-H4folate	465.30	313.20	70.0	−13.0	−20.0	−17.0	3.6–3.8
	465.30	180.15	70.0	−13.0	−37.0	−14.0	
	465.30	194.25	70.0	−23.0	−33.0	−22.0	
5-CHO-H4folate	474.30	327.15	70.0	−14.0	−20.0	−17.0	5.2–5.4
	474.30	299.20	70.0	−14.0	−31.0	−16.0	
	474.30	208.20	70.0	−18.0	−36.0	−24.0	
[13C5]-5-CHO-H4folate	479.25	327.15	70.0	−14.0	−20.0	−17.0	5.2–5.4
	479.25	299.20	70.0	−14.0	−31.0	−16.0	
	479.25	208.20	70.0	−18.0	−36.0	−24.0	
10-CHO-PteGlu	470.00	295.15	70.0	−23.0	−26.0	−22.0	5.0–5.2
	470.00	176.20	70.0	−23.0	−40.0	−20.0	
	470.00	120.15	70.0	−17.0	−39.0	−14.0	
MeFox	474.20	327.20	50.0	−16.0	−23.0	−24.0	4.3–4.5
	474.20	132.10	50.0	−16.0	−45.0	−24.0	
	474.20	284.15	50.0	−16.0	−37.0	−20.0	

References

1. Ringling, C.; Rychlik, M.; Origins of the difference between food folate analysis results obtained by LC-MS/MS and microbiological assays. *Anal. Bioanal. Chem.*, **2017**. *409(7)*, 1815–1825, doi: 10.1007/s00216-016-0126-4.
2. Striegel, L.; Chebib, S.; Netzel, M.E.; Rychlik, M.; Improved Stable Isotope Dilution Assay for Dietary Folates Using LC-MS/MS and Its Application to Strawberries. *Front. Chem.*, **2018**. *6(11)*, doi: 10.3389/fchem.2018.00011.
3. Vogelgesang, J.; Hädrich, J.; Limits of detection, identification and determination: A statistical approach for practitioners. *Accred. Qual. Assur.*, **1998**. *3(6)*, 242–255, doi: 10.1007/s007690050234.
4. Ahrens, H.; Mandel, John: The Statistical Analysis of Experimental Data. Wiley & Sons, Inc., New York 1964, 53 Abb., 109 Tab., 7 Taf. *Biom. J.*, **1968**. *10(4)*, 274-275, doi: 10.1002/bimj.19680100407.
